# Immediate transfection of patient-derived leukemia: a novel source for generating cell-based vaccines

**DOI:** 10.1186/1479-0556-3-4

**Published:** 2005-06-21

**Authors:** Jill A Gershan, Bryon D Johnson, James Weber, Dennis W Schauer, Natalia Natalia, Stephanie Behnke, Karen Burns, Kelly W Maloney, Anne B Warwick, Rimas J Orentas

**Affiliations:** 1 Department of Pediatrics, Medical College of Wisconsin and the Children's Research Institute, Children's Hospital of Wisconsin, 8701 Watertown Plank Rd., Milwaukee, WI 53226, USA

## Abstract

**Background:**

The production of cell-based cancer vaccines by gene vectors encoding proteins that stimulate the immune system has advanced rapidly in model systems. We sought to develop non-viral transfection methods that could transform patient tumor cells into cancer vaccines, paving the way for rapid production of autologous cell-based vaccines.

**Methods:**

As the extended culture and expansion of most patient tumor cells is not possible, we sought to first evaluate a new technology that combines electroporation and chemical transfection in order to determine if plasmid-based gene vectors could be instantaneously delivered to the nucleus, and to determine if gene expression was possible in a cell-cycle independent manner. We tested cultured cell lines, a primary murine tumor, and primary human leukemia cells from diagnostic work-up for transgene expression, using both RFP and CD137L expression vectors.

**Results:**

Combined electroporation-transfection directly delivered plasmid DNA to the nucleus of transfected cells, as demonstrated by confocal microscopy and real-time PCR analysis of isolated nuclei. Expression of protein from plasmid vectors could be detected as early as two hours post transfection. However, the kinetics of gene expression from plasmid-based vectors in tumor cell lines indicated that optimal gene expression was still dependent on cell division. We then tested to see if pediatric acute lymphocytic leukemia (ALL) would also display the rapid gene expression kinetics of tumor cells lines, determining gene expression 24 hours after transfection. Six of 12 specimens showed greater than 17% transgene expression, and all samples showed at least some transgene expression.

**Conclusion:**

Given that transgene expression could be detected in a majority of primary tumor samples analyzed within hours, direct electroporation-based transfection of primary leukemia holds the potential to generate patient-specific cancer vaccines. Plasmid-based gene therapy represents a simple means to generate cell-based cancer vaccines and does not require the extensive infrastructure of a virus-based vector system.

## Background

The efficacy of cell-based tumor vaccines in murine models of malignancy is well established. Using tumor cells lines transfected with soluble immune stimulatory molecules such as IL-2 or IL-12, or cell surface co-stimulatory antigens including CD80, and CD137L, or even allogeneic MHC results in profound immune activation [1-5]. The advantage of working in model systems is that unlimited amounts of tumor are available to produce cell-based vaccines. The ability to produce cell-based vaccines from clinic-derived material, however, remains a challenge.

Cell-based vaccines from tumor-derived material have been prepared and administered in either an allogeneic or autologous fashion, recently reviewed by Mocellin, *et al*. [1]. An allogeneic vaccine usually features the expansion of a single tumor cell line that can grow well in culture, genetic transduction by the desired vector, and preparation of large vaccine stocks that can be qualified for clinical use. A vaccine for neuroblastoma featuring the expression of both a cytokine and a chemokine transgene (IL-2 and lymphotactin) by a single human neuroblastoma cell line is a recent example of this strategy [7]. The disadvantage of a single cell line approach is that each malignancy is to some degree unique, and perhaps the most immunogenic antigens, or the most relevant ones for a given patient, will fail to be expressed by the allogeneic vaccine.

Give these limitations, we propose that a cell-based vaccine could be produced in an autologous manner for patients with a high disease burden, such as those who present with significant bone marrow involvement. For example, the large amount of tumor material typically available from leukemia patients makes these cells accessible for autologous patient-derived vaccine production.

A major hurdle to be overcome in using primary cells is the need to culture tumor cells *in vitro *in order for transduction or transfection procedures to be carried out. Most malignancies will not survive in culture in large enough numbers to be utilized. However, if the time required for *in vitro *manipulation was minimized, for example to 8–24 hours, patient-derived leukemia cells could be isolated from blood or bone marrow, transfected, and then upon irradiation used as a cell-based vaccine. Here we report the application of a novel electroporation-based transfection methodology that holds the potential to immediately transform a patient tumor sample into a cell-based cancer vaccine. This process, termed nucleofection, was pursued in our laboratory because it is the most rapid method of gene vector introduction available. We demonstrate that even though delivery of a plasmid gene vector to the nucleus is immediate, short-term culture is still required, and that a single-round of cell division may be needed to reach optimal gene expression levels. Importantly the time for tumor vaccine preparation is now measured in terms of hours instead of days. Our findings confirm studies carried out by Schakowski et al., where 3 samples from acute myeloid leukemia (AML) patients were transfected with a GFP expression vector [8]. The large degree of transgene expression in the majority of patient-derived acute lymphoblastic leukemia (ALL) specimens that we present here suggests that a clinical trial using these procedures should be pursued.

## Methods

### Cell lines

The mouse neuroblastoma cell line AGN2a, an aggressive subclone of Neuro-2a, was cultured in Dulbecco's modified Eagle's medium (DMEM), 100 U/ml penicillin, 100 μg/ml streptomycin, 2 mM L-glutamine and 10% heat inactivated fetal bovine serum (FBS), 1 mM MEM sodium pyruvate, 100 U/ml penicillin, 100 μg/ml streptomycin, 0.01 M HEPES buffer, 2 mM L-glutamine, 0.05 M 2-mercaptoethanol, and 0.069 M L-arginine HCl [4]. Primary murine tumor was generated by subcutaneous injection of 1 × 10^6 ^cultured AGN2a cells into strain A/J mice (Jackson Labs, Bar Harbor, ME). The human osteosarcoma cell line U2OS, kindly provided by Dr. Kent Wilcox, Medical College of Wisconsin, and the mouse squamous cell carcinoma cell line SCCVII, kindly provided by Dr. Scott Strome, Mayo Clinic, Rochester, MN, were cultured in DMEM as above. Mouse primary tumors were processed into single-cell suspensions by injection of 1–2 ml of 1 mg/ml collagenase D into the excised tumor mass (1 mg/ml in 10 mM HEPES, 150 mM NaCl, 5 mM KCl, 1 mM MgCl_2_, 1.8 mM CaCl_2_) and incubated at 37°C for 45 min followed by mechanical disruption through a sterile screen. Tumor cells were washed in DMEM and PBS and viable cells were separated by centrifugation over a Ficoll-Paque™ (Amersham Biosciences, Piscataway, NJ) density gradient.

## Transfection of tumor cell lines

Tumor cells were transfected with either pcDNA3.1/Hygro(-) (Invitrogen, Carlsbad, CA) or pDSRed2-C1 (BD Biosciences, San Diego, CA) plasmid vectors using a cationic lipid-based transfection methodology (Novafection, VennNova, Inc., Pompano Beach, FL) or a proprietary electroporation method (Nucleofection, Amaxa, Köln, Germany). Cells were nucleofected with 0.5 μg plasmid per 10^6 ^cells or lipid transfected with 0.5 μg plasmid and 2 μg of NovaFECTOR reagent per 10^6 ^cells. Similarly, U2OS, SCCVII and AGN2a cells were nucleofected with 0.5 μg per 10^6 ^cells pDSRed2-C1. To determine expression levels, cells were stained with 7AAD (BD Biosciences) and the expression of red fluorescent protein (RFP) in live gated cells was analyzed by flow cytometry (FACScan, Becton Dickinson, Franklin Lakes, NJ) at designated time points. U2OS and SCCVII cells were also nucleofected with pCI-neo (Promega, Madison, WI) encoding CD137L (4-1BBL) cDNA at a concentration of 1.5 μg per 10^6 ^cells [5]. The CD137L transfected cells were stained with phycoerythrin (PE) conjugated mouse anti-human CDw137 Ligand (BD Biosciences Pharmingen, San Diego, CA).

### Patient Samples

Patient leukemia and lymphoma samples were obtained in accordance to the Helsinki Declaration from excess bone marrow or peripheral blood specimens submitted to the Cell Marker Lab, Children's Hospital of Wisconsin, for routine leukemia screening. Studies involving these samples were approved by the Medical College of Wisconsin and Children's Hospital of Wisconsin Institutional Review Boards. Informed consent was obtained from the parents or guardians of each child and each sample was assigned a unique identifier number to ensure confidentiality.

### Transfection of primary acute lymphocytic leukemia cells

Leukocytes from bone marrow or peripheral blood patient samples were separated by centrifugation over a Ficoll-Paque™ density gradient. Cells were nucleofected with 1 μg pDSRed2C-1 (red fluorescent protein, RFP, expression vector) plasmid per 10^6 ^cells using a variety of Amaxa solutions and program parameters, cultured in RPMI-1640, 100 U/ml penicillin, 100 μg/ml streptomycin and 10% heat inactivated FBS for 24 hours then analyzed for RFP expression by flow cytometry (FACScan, Becton Dickinson). FACS acquisition and analysis was done using either propidium iodide (PI) or 7AAD to exclude dead cells. The leukemic blast population phenotype was determined by the flow cytometric and CD antigen expression profile as compared to normal cell populations. Both CD45+ and CD45- leukemic blasts could be gated when stained with anti-CD45 antibody and analyzed by flow cytometry for CD45 expression and side scatter properties. All antibodies utilized were clinical grade direct fluorochrome conjugates (Becton Dickinson).

### Confocal microscopy

U2OS cells were nucleofected with 3 μg fluorescein labeled (Mirus *Label *IT^® ^Tracker, Madison, WI) pUC19 plasmid or pDSRed2C-1 plasmid per 10^6 ^cells. Immediately, or 3 days following nucleofection, cells were washed in cDMEM and counted. Cells were fixed on a glass slide with 3.7% buffered formalin, washed, permeabilized with 0.5% Triton X-100 (Surfact-amp, Pierce, Rockford, IL) and washed again. Pearmeabilized cells were incubated with 2.4 nM TOTO3 (Molecular Probes, Eugene, OR) and washed. Vectashield (Vector Laboratories, Inc., Burlingame, CA) was added to the cells prior to sealing with a coverslip. Optical sectioning of cells was taken sequentially using argon (488 nm excitation) and helium/neon (633 nm excitation) lasers on a Leica SPT S2 confocal microscope with a 100x oil immersion lens.

## Quantitative real-time PCR

U2OS cells were nucleofected with 0.5 μg pDSRed2C-1 plasmid per 10^6 ^cells. Cells were harvested and used for nuclear DNA isolation. Prior to DNA isolation, nuclei were washed in PBS and incubated with 0.5U DNase I (Ambion, Austin, TX) at 37° for 10 min and washed again twice in PBS. Nuclear DNA was isolated (Nuclei EZ prep, Sigma, Saint Louis, MO) from transfected cells at designated time-points. The plasmid encoded neomycin phosphotransferase gene (*neo*) was amplified with primers and TaqMan hydrolysis probe as described by Sanburn, et al. [9]. Nuclear DNA from each of three experimental and three parallel control samples (U2OS cells Nucleofected without plasmid) was amplified in triplicate in an Opticon™ 2 Cycler (MJ Research™, Inc., Waltham, MA) with the following cycling protocol: 50°C 2 min, 95°C 10 min, with 40 cycles of 95°C for 15 sec., and 62°C for 1 min. To normalize the number of cells/nuclei, human RNase P was amplified using the TaqMan^® ^RNase P reagents kit (Applied Biosystems, Foster City, CA), or for mouse cells, mouse Apo B was amplified using the primers 5' CACGTGGGCTCCAGCATT 3'and 5' TCACCAGTCATTTCTGCCTTTG 3' and the TaqMan hydrolysis probe 5'(FAM) CCAATGGTCGGGCACTGCTCAATA (TAMRA) 3' (courtesy Renee Horner, qpcrlistserver, yahoo groups, yahoo.com). The *neo *gene copy number per cell was determined using a plasmid-based standard curve.

## Analysis of cell division

Cells were suspended in PBS and incubated with CFDA SE (5-(and -6)-carboxyfluorescein diacetate succinimidyl ester, CFSE (Molecular Probes, Eugene, OR) at a final concentration of 0.35 μM per 4 × 10^6 ^cells, incubated for 10 min at 37°C, and washed x3 in DMEM, 10% FBS. CFSE expression was analyzed by flow cytometry to assess cell division.

### Cell cycle blockade

At 50–60% confluency, 0.6 mM mimosine (Sigma, Saint Louis, MO) was added to U2OS cells (2). Both U2OS and U2OS cells treated with mimosine were incubated at 37°C for 48 hours at which time the cells were harvested, counted and nucleofected with 1.5 μg per 10^6 ^cells pCI-neo vector encoding human 4IBBL (CD137L) cDNA [5]. As a control, cells were also nucleofected without plasmid. Four hours post-nucleofection cells were harvested, counted, stained with phycoerytherin (PE) labeled anti-human CD137L (BD Biosciences) and 7AAD (BD Biosciences), and analyzed for CD137L-expression by flow cytometry. To determine DNA content prior to nucleofection, cells were washed in phosphate buffered saline (PBS), fixed with 4% paraformaldehyde, washed again in PBS, and 1 μl propidium iodide (BD Bioscience) at 50 ug/ml was added. The propidium iodide labeled cells were then analyzed by flow cytometry [11].

## Results

### Optimization of Transfection by Electroporation

**Figure 1:**
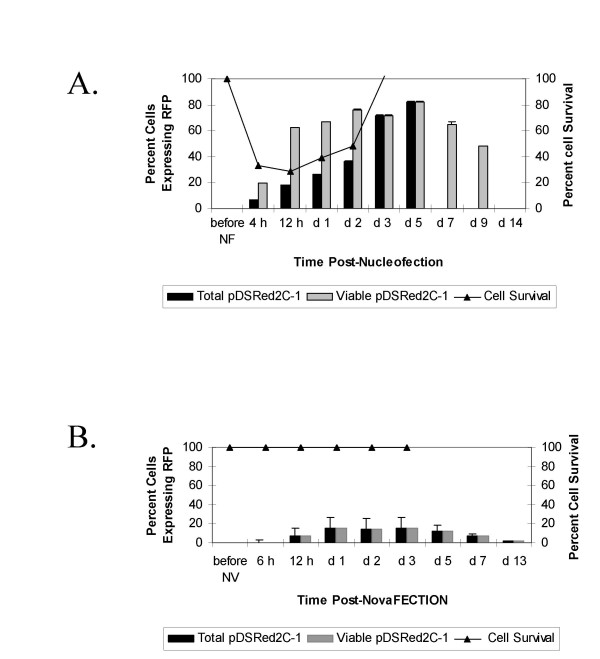
Kinetics of transgene expression in electroporated and cationic lipid transfected U2OS cells. (A) RFP expression over time in cultured U2OS cells when nucleofected with pDSRed2C-1 (RFP) plasmid. Black bars represent the percent cells expressing RFP corrected for the total number of cells transfected and gray bars represent the percent of viable cells expressing RFP. (B) RFP expression over time in cultured U2OS cells when novafected with pDSRed2C-1 (RFP) plasmid. Since there is no cell death associated with novafection, the gray and black bars both represent the percent cells expressing RFP from the total number of cells transfected. Autofluorescence (always <2%) detected from cells nucleofected or novafected with pcDNAHygro(-) control plasmid was subtracted from the experimental values. The black line in both A and B represents average cell number at each time point. All experiments were done in triplicate with the standard deviation indicated by the error bars.

To determine differences in the kinetics and strength of expression of a transfected reporter gene using either an electroporation method (nucleofection) or a cationic liposome-based methodology (novafection), U2OS human osteosarcoma cells were transfected with 0.5 μg pDSRed2C-1 (RFP) plasmid per 10^6 ^cells, and RFP protein expression measured over time by flow cytometry. Following nucleofection, RFP was expressed as early as 4 hours in U2OS cells, Figure 1A. By 12 hours, 60% of the nucleofected cells expressed RFP. When this rate was adjusted for the cell death associated with nucleofection, the transfection rate dropped to 18% of the total cells initially transfected now expressing RFP at 12 hours. On day 3, when the percent viability of the nucleofected cells returned to 100%, the transfection rates no longer needed correction and the reported rates are identical. This expression was maintained for several days and gradually diminished until day 14 when expression could no longer be detected. In contrast, using novafection, RFP expression required 12 hours of culture (as opposed to 4 hours for nucleofection) and did not approach peak expression levels until day 1, Figure 1B. The efficiency of transfection was determined by using identical amounts of plasmid gene vector in each method. Upon comparison of RFP expression levels at day 1, the superiority of electroporation-based transfection was evident. In the viable fraction of nucleofected cells, 60% of these cells expressed RFP at 24 hours, as opposed to 15% of the novafected cells. Even when corrected for cells lost to electroporation-associated cell death, the nucleofection expression rate is 26%. As a control, cells were also nucleofected with a non-RFP expressing plasmid, pcDNA3.1/Hygro(-), and the minimal autofluoresence of transfected cells (less than 2%) subtracted from the reported expression levels. The RFP expression levels in cells transfected with the liposome-based reagent could be increased by increasing the amount of plasmid DNA, therefore these comparisons are relative and not maximized for each method (not shown).

**Figure 2:**
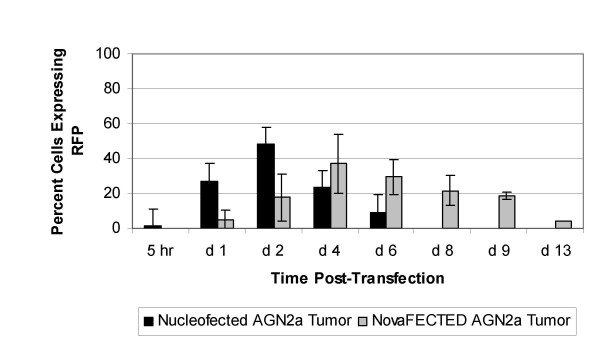
Kinetics of transgene expression in electroporated and cationic liposome transfected mouse primary tumor. The percent AGN2a mouse primary tumor cells expressing RFP in nucleofected (black bars) and novafected (gray bars) cells is shown. Autofluorescence detected from cells nucleofected or novafected with pcDNAHygro(-) control plasmid was subtracted from the experimental values. All experiments were done in triplicate with the standard deviation indicated by the error bars.

**Figure 3:**
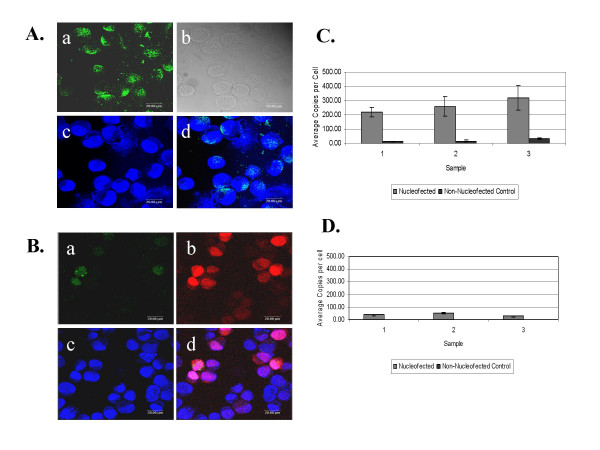
Confocal images of FITC-labeled plasmid in U2OS cells immediately and 3 days following nucleofection. A) Confocal images captured immediately following nucleofection: a) fluorescence of FITC-labeled pUC19 plasmid, b) phase contrast image, c) TOTO3 stained nuclei (dark blue), d) overlay of the images in (a) and (c) showing FITC-labeled plasmid in the nucleus. B) Confocal images captured 3 days following nucleofection: a) fluorescence of FITC-labeled pDSRed2C-1 plasmid, b) fluorescence from expression of RFP encoded on the FITC-labeled pDSRed2C-1 plasmid, c) TOTO3 stained nuclei, d) an overlay of the images in a, b and c showing FITC-labeled plasmid, expression of RFP and nuclear localization. C) Average number of plasmid copies per cell as determined by real-time PCR of the plasmid encoded *neo *gene from nuclear DNA harvested immediately following nucleofection. D) Plasmid *neo *copies amplified from nuclear DNA harvested 3 days following nucleofection. In both C and D the gray bars represent *neo *amplification from U2OS cells nucleofected with 0.5 μg pDSRed2C-1 per 10^6 ^cells and the black bars represent *neo *amplification from non-nucleofected U2OS cells. Nuclear DNA was harvested and amplified from 3 separate samples, error bars indicate the standard deviation from the average of triplicate samples.

We then tested a primary mouse-derived neuroblastoma mass for the ability to be transfected by these methodologies. AGN2a tumor cells were injected subcutaneously into host strain mice, A/J, and the resulting subcutaneous tumor cell mass was excised, processed into a single-cell suspension, transfected with 0.5 μg pDSRed2C-1 (RFP) plasmid per 10^6 ^cells, and RFP expression over time measured by flow cytometry. Nucleofected primary murine tumor cells began to express RFP earlier than novafected cells (5 hr versus 1 day) and expression levels peaked at day 2 in viable nucleofected cells and day 4 in novafected cells, Figure 2. This later peak is likely due to the longer duration of transgene expression in novafected cells. Both the kinetics and total RFP expression levels differed for the human U2OS cell line, Figure 1, and the primary mouse-derived tumor, AGN2a, Figure 2. The nucleofection rates were not as high for the nucleofected primary tumor, while the novafection rate improved. These are vastly different systems and the rapid cell division rate of the cultured U2OS, Figure 3, as opposed to uncultured tumor that was excised, processed into a single cell solution and then transfected, may partially explain this result.

The primary limitation of electroporation-based transfection is cell death. Preliminary experiments confirmed that increasing the strength of the electric field corresponded to both a higher transfection rate, and increased cell death. The nucleofection setting that we found optimal resulted in 70% cell death, Figure 1A. Cell numbers gradually recovered post-nucleofection, beginning at 24 hours. In contrast, there was no decrease in cell number following novafection, Figure 1B.

### Delivery of plasmid DNA to the nucleus by electroporation is rapid and short-lived

The inability to culture most primary human tumors led us to search for methods of transfection that would require minimal culture and processing time while allowing for efficient gene transfection. Given the rapid kinetics of expression using nucleofection, we sought to determine if this was due to direct delivery of plasmid DNA in to the nucleus. Confocal microscopy was used to visualize individual z-plane sections that represent internal nuclear layers of U2OS cells that had been nucleofected with 3 μg FITC-labeled pUC19 plasmid per 10^6 ^cells, immediately cytospun onto glass slides, and then prepared for microscopy. The nuclear and cytoplasmic boundaries of nucleofected cells were visualized by phase contrast microscopy, Figure 3A, panel b, or by staining with the nuclear dye TOTO3, Figure 3A, panel c. The nuclei are stained dark blue, with a lighter blue staining in the cytoplasmic compartment. The plasmid-associated fluorescein signal was present in both the cytoplasmic and nuclear compartments immediately following nucleofection, Figure 3A, panel d. Visual inspection reveals that most cells contained nuclear plasmid, Figure 3A, d (an overlay of the plasmid signal with the TOTO3 stain).

Using the same technique, we then sought to determine how long after nucleofection the plasmid vector remained in the nucleus. Three days after nucleofection of U2OS cells with 3 μg FITC-labeled pDSRed2C-1 (RFP) plasmid per 10^6 ^cells, the presence of plasmid vector DNA, was greatly diminished, Figure 3B, panel a. The presence of plasmid vector DNA, as detected by FITC fluorescence, was seen in a small minority of cells, and when present on day 3 it appeared to associate more with a punctate fluorescence in the cytoplasm, Figure 3B, a and d. Despite the loss of plasmid vector from the nucleus, intense red fluorescence was seen in many of the cells at this time, indicating the continued presence of red fluorescent protein, Figure 3B, panels b and d.

To further confirm the presence of plasmid in the nucleus, the copy number of plasmid vector per cell was calculated by real-time PCR amplification of the pDSRed2C-1 encoded neomycin phosphotransferase gene (*neo*). U2OS cells were nucleofected with 0.5 μg pDSRed2C-1 per 10^6 ^cells and immediately, or at day 3, nuclear DNA was isolated from the nuclear fraction of cell lysates and PCR amplified using *neo *primers and a *neo-*specific TaqMan probe. The total number of plasmid *neo *copies was calculated based on comparison to a standard curve generated with the same plasmid vector. The number of cells (or nuclei) analyzed was determined using a standard curve calibrated to genomic DNA mass and signal from the single copy gene *RNAseP*. Nuclei were purified on a sucrose cushion, washed with PBS, digested with DNAse in order to remove contaminating cytoplasmic plasmid DNA, and total DNA extracted. Immediately following nucleofection, there were 200 to 400 copies of plasmid per cell, Figure 3C. In agreement with microscopy data, copy number in U2OS cell nuclei decreased to 50 copies or less per cell by day 3 post-nucleofection, Figure 3D. Immediate localization of plasmid to the nucleus following nucleofection was also observed by real-time PCR in the AGN2a and SCCVII cell lines (data not shown).

### Delivery of plasmid gene vectors to the nucleus requires cell division for optimal gene expression

**Figure 4:**
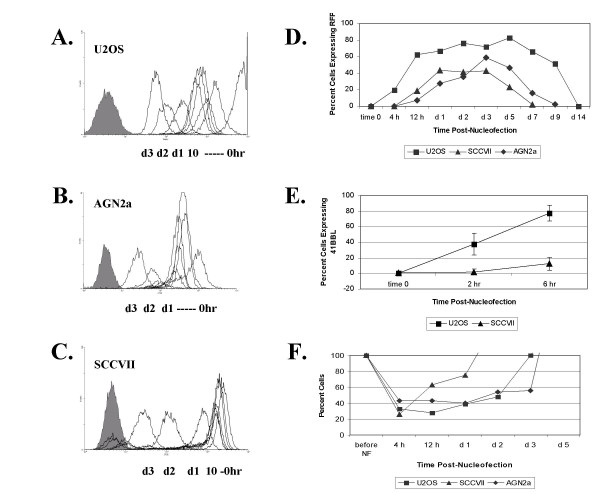
Cell division and gene expression in U2OS, AGN2a and SCCVII cells. Tumor cells were labeled with CFSE to detect changes in fluorescence that occur with cell division post-nucloefection. (A) U2OS, (B) cultured AGN2a, (C) SCCVII cells were analyzed for CFSE fluorescence by flow cytometry immediately following nucleofection or cultured up to 3 days following nucleofection. The solid gray peak (far left of each panel) represents unstained cells. Peaks decreasing in fluorescence (right to left) indicate the loss of CFSE fluorescence over time as cells divide. Cells were analyzed at hour 2, 4, 6, 8, 10, day 1, day 2, and day 3. Only U2OS showed peaks indicating decrease in fluorescence prior to day one: at hour 2, and hours 4–10. D) In parallel experiments, the average percent of cells expressing RFP from 3 separate experiments at the indicated time points following nucleofection was determined in U2OS (■), SCCVII (▲) and AGN2a (◆) cell lines by flow cytometry. E) Average percentage of cells expressing CD137L at time 0, 2 hours, and 6 hours post-Nucleofection in U2OS and SCCVII cells. The error bars represent the standard deviation from 3 experiments. F) Average viable cell number of U2OS, SCCVII and AGN2a tumor cells post-nucleofection from 3 separate experiments, expressed as a percentage of the number of cells originally nucleofected.

The ideal cell-based cancer vaccine would allow recombinant gene expression in primary human tumor cells. The bottleneck is that primary human tumors do not grow and divide efficiently in culture. Therefore, it was essential to evaluate the association between cell division and expression of nucleofected genes. To determine the pattern of cell division rates in nucleofected tumor cell lines (U2OS, AGN2a and SCCVII), cultured tumor cell lines were incubated with carboxyfluorescein diacetate succinimidyl ester (CFSE). The intensity of CFSE fluorescence was analyzed by flow cytometry every two hours for 10 hours and then daily for 3 days after nucleofection, Figure 4A–C. According to the decrease in CFSE fluorescence intensity with each cell division (leftward shift of peaks), the U2OS cells underwent about 3 cell divisions in the first 10 hours post-nucleofection, whereas, the AGN2a and SCCVII cells divided approximately once in the first 10 hours post-nucleofection. CFSE stained cells were also nucleofected with RFP-encoding plasmid and there was no difference in the CFSE fluorescent pattern by flow cytometry in the nucleofected versus non-nucleofected cells (data not shown). As demonstrated previously, it was the rapidly dividing U2OS cells that exhibited transgene expression at 4 hours (20% of the viable expressed RFP), while RFP could not be detected in the slower dividing AGN2a and SCCVII until 12 hours post-nucleofection, Figure 4D.

To further explore this finding, we used an alternate plasmid-encoded transgene and compared the kinetics of expression in rapidly and slower dividing cell lines. Our laboratory has produced a number of immune co-stimulatory expression vectors, and we chose a human CD137L (4IBBL) expression vector for further study. Expression of this cell-surface antigen can be directly measured by flow cytometry using a labeled CD137L-specific antibody. One of our concerns with using RFP was that the DsRed2 protein we utilized requires approximately 6 hours to reach full fluorescence intensity due to a requirement for intracellular oxidation [12]. Therefore a CD137L-encoding plasmid, 1.5 μg per 10^6 ^cells, was nucleofected into the U2OS and SCCVII cells and expression compared. In the rapidly dividing U2OS, 40% of the cells expressed CD137L as early as 2 hours post-nucleofection. At 6 hours post-nucleofection, 80% of the U2OS cells expressed CD137L, Figure 4E. In contrast, only 10% of SCCVII cells expressed CD137L at 6 hours post-nucleofection. The nucleofection process also induced significant cell death, demonstrating that cell death was not an RFP-associated phenomenon, Figure 4F. All cells experienced 60 to 80% cell death upon nucleofection, however the SCCVII cells recovered much more rapidly than either the rapidly dividing U2OS cells or the less rapidly dividing AGN2a cells, indicating that factors other than cell division are involved in cellular recovery from the electroporation and transfection processes.

**Figure 5:**
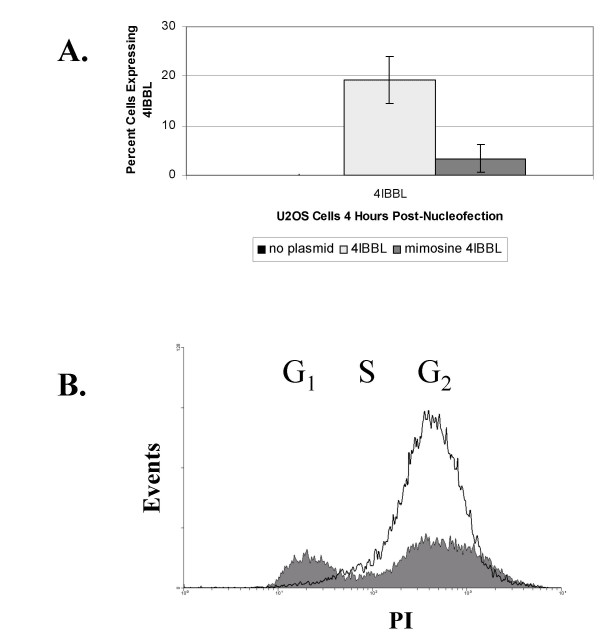
Impact of cell cycle inhibition on gene vector expression. A) The average percentage of U2OS cells expressing vector-encoded CD137L 4 hours following nucleofection. The black bar represents cells nucleofected without plasmid, stippled bar represents untreated cells, and the gray bar represents mimosine treated cells. The error bars show the standard deviation from 3 separate experiments. B) Flow cytometric profile of propidium iodide stained mimosine treated U2OS cells (solid gray) and mimosine untreated U2OS cells (black line) prior to nucleofection of CD137L.

To directly assess the need for cell division, U2OS cells were incubated with 0.6mM mimosine, which blocks cell cycle progression, and CD137L expression of nucleofected cells compared to non-treated controls, Figure 5A. In non-mimosine treated U2OS cells, approximately 20% of cells expressed CD137L at 4 hours post-nucleofection. In contrast, less than 4% of mimosine treated cells expressed CD137L at 4 hours post-nucleofection. To assure that cell cycle block occurred in mimosine treated cells, cell cycle status was determined by propidium iodide staining. The majority of mimosine treated cells were in G1/G0 while the mimosine untreated cells had a greater proportion of cells in S, and G2/M phases of the cell cycle, Figure 5B. This finding indicates that cell cycle progression enhances transgene expression by nucleofection. This finding was unexpected as it implies that other mechanisms are at work besides the delivery of plasmid DNA to the nucleus.

### Application of electroporation-based transfection to human leukemias

To assess the ability of electroporation-based transfection to be utilized in the production of autologous tumor vaccines for patients, we first determined the optimal electrotransduction parameters. Fresh primary leukemia cells from 6 patient samples were nucleofected with 1 μg of pDSRed2C-1 plasmid per 10^6 ^cells, using three different Amaxa solutions (R, T, V) and several different electrotransduction programs (T20, U15, T17, T27, S04, O17, T16, T01, and O17). The expression of RFP protein was determined by flow cytometry 24 hours post-nucleofection. There was a wide range of RFP expression depending on the electroporation conditions, Table 1. Amaxa solution R with program T20 and solution T in combination with program U15 consistently provided the highest expression of RFP protein. The highest expression level seen was 62%, (sample 4 PB) and the lowest expression was 1.8% (sample 12 BM). The RFP expression of 12 total samples collected using solution R and program T20, along with available diagnostic phenotypic information, is presented in Table 2. The R/T20 settings were chosen based on a slightly better viability profile than the T/U15. Greater than 5% expression was seen in 9 of 12 samples and greater than 20% expression was seen in 5 of 12 samples. These patient leukemias were extensively characterized for standard childhood leukemia markers, and no phenotype was clearly associated with the ability to be transfected using the parameters we established. Although not reported, the number of leukemic cells obtained per patient was variable- as the samples obtained for this study were essentially diagnostic remains that were to be discarded. For two of the samples (5 and 6) we had both peripheral blood and bone marrow-derived matched samples. In both cases the peripheral blood cells showed a higher transfection rate. Our certainty that the transfected cells analyzed were the leukemia cells comes from the clinical diagnostic experience of the Cell Marker Laboratory of the Children's Hospital of Wisconsin, where the identical procedures used for clinical diagnosis of malignancy were used to analyze the nucleofected samples. The majority of the samples we analyzed were bone marrow aspirates. In patients with advanced disease, autologous bone marrow may prove to be both an accessible and abundant source of tumor cells that can be modified by plasmid-based gene vectors to produce cell-based vaccines.

**Table 1:** Comparison of nucleofection parameters in ALL patient samples. Nucleofection using an RFP expression vector was carried out using 6 different patient samples (patients 4,13,8,11,19,12) from one of two potential tissue sources, PB, peripheral blood, or BM, bone marrow. ^1^No NF, not nucleofected autofluorescence control. ^2^Nucleofection was carried out with one of three solutions R, T, or V, and the following electrical settings on the Amaxa nucleofection device: T20, U15, T17, T27, S04, O17, T16, T01, O17. ^3^The table lists the percentage of viable cells expressing the plasmid-encoded RFP at 24 hours post-nucleofection.

Electroporation Parameters										
Patient Samples	No NF^1^	R^2^/T20	T/U15	R/T17	R/T27	T/S04	T/O17	R/T16	V/T01	V/O17

4 PB	^3^0.1	62.3	61.5	56.8	52.9	52.5	46.2	46.1	40.5	20.9
13 BM	1.0	45.0	45.6	42.9	40.2	32.4	36.1	38.5	21.3	36.1
8 PB	1.0	9.2	10.9	9.3	6.7	8.2	5.6	6.4	6.1	3.7
11 BM	1.2	17.1	25.2	13.2	13.4	9.5	5.7	10.0	5.7	7.1
10 BM	0.1	8.0	10.0	4.6	4.5	2.3	2.0	3.9	2.0	0.9
12 BM	1.3	2.9	3.6	2.4	2.3	2.6	3.5	1.8	2.1	3.6

## Discussion

Cell-based autologous cancer vaccines hold great promise in the effort to shift the adaptive immune system from ignorance or anergy toward cell-mediated immune recognition of cancer. The generation of a Th1-type immune response with cell-based vaccines, and the resultant CTL-mediated killing of tumor, have offered the most effective anti-tumor responses in pre-clinical models, and also initiated clinical responses, as reviewed by Nitti et al., [13]. *Ex vivo *introduction of gene vectors encoding immune activating signals (such as co-stimulatory antigens, cytokines and adhesion molecules) into tumor cells with subsequent re-introduction of irradiated modified tumor cells into patients is currently being pursued with the aim of initiating a clinically-relevant immune response against tumor [14,15]. Because this type of therapy involves cell processing, culture procedures, as well as genetic transfer, any steps that streamline the process will make a significant contribution toward tumor vaccine development.

Newer generation transfection procedures based on electroporation or branched polyethyleneamine (PEI) have been reported to be independent of cell cycle effects [14]. Nevertheless, we found that cell cycle progression does enhance transgene expression and that certain tumor cell lines (such as U2OS) thought to be highly "transfectable" may be highly amenable to vector-derived transgene expression precisely because they have rapid rates of cell division. Most reports do not analyze transgene expression at the early times (2–8 hours) we report here, and unless proven otherwise, the contribution of cell cycle progression within the first 8 hours of transfection cannot be excluded. We examined this question in detail because our goal is to transfect primary human leukemias that do not persist in tissue culture and thus have limited *in vitro *proliferative capacity.

Having thus defined the problem, we examined in detail a transfection procedure that appears to be the most rapid. Nucleofection delivers plasmid vector DNA in to the nucleus immediately, Figure 3. However, as we demonstrate here, nucleofection still benefits from cell cycle progression and cell division, Figures 4,5. Using nucleofection we found that tumor cells harvested directly from a mouse bearing the AGN2a neuroblastoma could be nucleofected with high efficiency. Thus, the process of tumor disaggregation and processing did not render the neuroblastoma cells we studied resistant to nucleofection. Confident of these results, we initiated analysis of primary human tumors.

The tumor samples we analyzed were from patients undergoing diagnosis for suspected childhood leukemia. If samples from blood or bone marrow remained after diagnostic procedures were completed, they were then tested for the ability to be nucleofected. Nine of 12 specimens showed greater than 5% transgene expression, and half of these showed greater than 17% expression, Table 2. This means that for the majority of the patients we tested, nucleofection with transgenes that encode either immune co-stimulatory molecules or cytokines may be feasible. We did not analyze gene expression in nucleofected ALL specimens over time, as our long-term goal is to transfect and then use these cells as experimental vaccines soon thereafter. Studies with other cultured tumor cell lines in our lab have shown that transgene expression rarely persists beyond 9 days, as nucleofected plasmid vectors do not integrate into the genome. While clinical samples could be frozen, thawed, cultured overnight, and then nucleofected, the viability of the frozen and thawed clinical samples was low, between 10–25% (not shown). We propose that the highest nucleofection rates are likely to be seen using fresh leukemic cells, and that viability upon thawing may not be critical as cells will be irradiated prior to use as a vaccine. Future studies will be carried out to address this issue. Our report also highlights that the nucleofection of primary ALL was quite variable. Reasons for this could be due to a specific phenotype that could be identified by expression profiling. Building upon our *in vitro *data it may be the ability of one clinical sample to undergo cell division or even limited expansion in culture over another during the overnight culture period following nucleofection may allow for better transgene expression. Thus, inclusion of growth factors or cytokines may increase nucleofection rates above those we report here.

**Table 2:** Transfection of Patient Leukemia. Patient leukemia cells were nucleofected with solution R, setting T20, as described in Methods. Patient diagnosis and specimen type are listed (PB, peripheral blood, BM, bone marrow). Relevant diagnostic criteria with respect to phenotype is also listed. Transfection efficiency is based upon the percentage of viable cells expressing RFP at 24 hours post-nucleofection.

**Patient Sample**	**Diagnosis**	**Specimen**	**Phenotype**	**Transfection Efficiency**
4	ALL	PB	45+/Tdt+/DR+/34+/10-/19+/20–24+/9+/56+	62.3
13	ALL	BM	45+/Tdt+/DR+/34-/10+/19+/20+/24+/9-/52+/22+	45.0
14	ALL	BM	45-/Tdt+/DR+/34–10+/19+/20dim/24+/9+	44.3
9	ALL-CD56	BM	45-/Tdt+/DR-/34+/10+/19+/20-/24-9+/56+	26.3
5	ALL-CD33	(a)PB (b)BM	45+/Tdt+/DR+/34+/10+/19+/20–24+/9+/33+/117dim	23.0 (a) 13.8 (b)
11	ALL	BM	45+/Tdt+/DR+/34+/10+/19+/20-/24+/9+/13-/33-/117-/52+/25+	17.1
8	ALL	PB	45+/Tdt+/DR+/34-/10+/19+/20het/24+/9+/μ-/κ-/λ-	9.2
10	ALL-CD13/33	BM	45-/Tdt+/DR+/34+/10+/19+/20-/24+/9+/13+/33+/117-	8.0
3	ALL-CD15	BM	45-/Tdt+/DR-/34+/10-/19+/20-/24-9+15+/56+	5.9
7	ALL	BM	45-/Tdt+/DR+/34+/10+/19+/20–24+/9+	4.3
6	ALL	(a)PB (b)BM	45-/Tdt+/DR+/34+/10+/19+/20-/24-/9+	3.4 (a) 1.0 (b)
12	T-ALL	BM	45+/Tdt+/DR+/34+/10-/19-/20-/24-/9-/13-/33-/117-/52+/7+/5+/2+/3-/4+/8-/1a-/TCRab-/ TCRgd-	2.9

## Conclusion

The process of nucloefection delivers plasmid DNA directly to the nucleus. Even though delivery to the nucleus is thought to circumvent dependence on cell division, we found that the highest and earliest levels of transgene expression from plasmid-based vectors occurred in rapidly dividing cells. We were also able to demonstrate that primary acute lymphocytic leukemia cells (ALL) from pediatric patients could also be nucleofected with plasmid-based vectors, thus opening the door to patient-specific cell manipulation. In light of the laboratory studies we present, transfection rates of clinical samples may be increased even further if some degree of cell division could be induced during the *in vitro *culture of these specimens. Even though this culture time is less than 24 hours, a single cell division could potently increase transgene expression levels, and thus immunogenicity of the vaccine preparation. Future work will include analysis of the correlation between the ability to be nucleofected, markers of cell cycle progression, and the induction of cell cycle progression post-nucleofection.

## Declaration of competing interests

The author(s) declare that they have no competing interests.

## Authors' contributions

JG, BJ, SB and JW carried out laboratory studies on established cells lines. JG carried out all confocal microscopy. DS analyzed marker expression on clinical samples and organized clinical data, NN was responsible for transfection of clinical samples, KB, KM, and AW were responsible for clinical care, patient consent, and pathological interpretation, RO was responsible for study design and execution.
